# Effects of indirect actions and oxygen on relative biological effectiveness: estimate of DSB induction and conversion induced by gamma rays and helium ions

**DOI:** 10.1093/jrr/rrv025

**Published:** 2015-04-22

**Authors:** Ju-Ying Tsai, Fang-Hsin Chen, Tsung-Yu Hsieh, Ya-Yun Hsiao

**Affiliations:** 1 Institute of Biotechnology and Department of Life Science, National Tsing Hua University, Hsinchu, Taiwan, Republic of China; 2 Department of Medical Imaging and Radiological Sciences, Chang Gung University, Kweishan, Taiwan, Republic of China; 3 Radiation Biology Research Center, Institute for Radiological Research, Chang Gung University/Chang Gung Memorial Hospital, Linkou, Taoyuan, Taiwan, Republic of China; 4 Department of Medical Imaging and Radiological Sciences, Chung Shan Medical University, No. 110, Section 1, Chien-Kuo N Road, Taichung, 402, Taiwan, Republic of China

**Keywords:** base excision repair, indirect action, relative biological effectiveness, clustered DNA damage, enzymatic DSB

## Abstract

Clustered DNA damage other than double-strand breaks (DSBs) can be detrimental to cells and can lead to mutagenesis or cell death. In addition to DSBs induced by ionizing radiation, misrepair of non-DSB clustered damage contributes extra DSBs converted from DNA misrepair via pathways for base excision repair and nucleotide excision repair. This study aimed to quantify the relative biological effectiveness (RBE) when DSB induction and conversion from non-DSB clustered damage misrepair were used as biological endpoints. The results showed that both linear energy transfer (LET) and indirect action had a strong impact on the yields for DSB induction and conversion. RBE values for DSB induction and maximum DSB conversion of helium ions (LET = 120 keV/μm) to ^60^Co gamma rays were 3.0 and 3.2, respectively. These RBE values increased to 5.8 and 5.6 in the absence of interference of indirect action initiated by addition of 2-M dimethylsulfoxide. DSB conversion was ∼1–4% of the total non-DSB damage due to gamma rays, which was lower than the 10% estimate by experimental measurement. Five to twenty percent of total non-DSB damage due to helium ions was converted into DSBs. Hence, it may be possible to increase the yields of DSBs in cancerous cells through DNA repair pathways, ultimately enhancing cell killing.

## INTRODUCTION

When cells are irradiated with ionizing radiation, both direct and indirect actions of the radiation may result in DNA damage [1]. Among this radiation damage, double-strand breaks (DSBs) are the most important form of DNA damage, and their misrepair results in mutation, cell death and transformation [2]. The indirect actions also affect DSB yields and the types of damage induced; however, their contributions generally decrease as linear energy transfer (LET) increases. Studies show that DNA damage is spatially denser for higher-LET radiation as compared with that caused by low-LET radiation: this dense damage is more difficult to repair [3, 4]. Previous experimental studies have also shown that repair efficiency for dense damage is lower than that for simple or individual damage [5–8].

Oxygen concentration is another important factor affecting DSB yields, especially those due to indirect action. Free radicals react with oxygen, transforming to RO_2_, which initiates a chain of events that finally results in DNA damage [1]. The DNA damage can be repaired under hypoxia, but may be fixed and irreparable if molecular oxygen is present. The biological effects of oxygen concentrations on cells can be quantified in terms of the oxygen enhancement ratio (OER), the ratio of hypoxic dose to aerated dose needed to achieve the same biological effects [9]. The OER for DSB induction ranges from 3 to 1.4 when the LET increases from 1 to 140 keV/μm. Its trend is opposite to that of the relative biological effectiveness (RBE) [10], suggesting that oxygen plays an important role in RBE as estimated by the number of DSBs. In addition to the DSB yields induced directly or indirectly by ionizing radiation, enzymatic DSB is another contributor to DSB yields [10]. Repair of non-DSB clusters, such as base damage (BD) and complex single-strand breaks (SSBs) with BD, proceeds mainly through the base excision repair (BER) pathway [11, 12], in which short-patch (SP) and long-patch (LP) repairs are involved [13]. In most cases of the SP BER pathway, single-nucleotide replacement occurs [14]. In contrast, LP BER removes a fragment of 2–13 nucleotides [15]. However, non-bulky lesions such as BD and oxidative DNA lesions, which are ignored by the BER pathway, can be processed by the nucleotide excision repair (NER) pathway [16, 17]. NER is a primary pathway that removes UV-induced bulky photoproducts [18] and is involved in the removal of free-radical–induced cyclodeoxynucleosides in mammalian cells [19, 20]. Non-DSB clusters that remain misrepaired after processing by BER and NER may lead to complex DNA clusters and undergo conversion into DSBs mediated by BER- and NER-associated enzymes (termed ‘enzymatic DSBs’) [21–23]. Most studies on RBE focus on DSB induction by ionizing radiation (termed ‘prompt DSBs’), but the biological effects of enzymatic DSBs are getting noticed [24–26]. Misrepair of non-DSB clustered damage via either the BER or NER pathway may extend the lifetime of these lesions in cells, increasing the likelihood of their conversion to enzymatic DSBs. Monte Carlo simulations have shown that the yields of enzymatic DSBs could reach 63% of those of prompt DSBs due to 1-MeV electrons [27], suggesting that enzymatic DSBs may be an important source of DSBs.

Recently, Bajinskis found that BER pathway plays an important role in the repair of DNA lesions induced by low-LET radiation [28]. The use of dimethylsulfoxide (DMSO) as a free radical scavenger reduced the number of unrepaired DSBs and increased cell survival, indicating that indirect action increases the complexity of DNA damage [28, 29]. Yet it is unclear how indirect action affects repair outcomes via BER pathways, as these cells are involved in several repair pathways. Likewise, obtaining experimental data for repair outcomes of a particular type of DNA lesion such as SSB or BD is difficult. In this study, we simulated the repair outcomes of the BER or BER/NER pathways under different damage types and repair scenarios. We also investigated the properties of prompt DSBs induced by ionizing radiation and enzymatic DSBs converted from misrepair. Furthermore, we analyzed the effect of oxygen concentration on the yields of non-DSB clustered damage due to low- and high-LET radiation. Our results showed that the RBE values for prompt DSBs and enzymatic DSBs increased significantly because of the absence of indirect actions of both low- and high-LET radiation. In addition, oxygen increased the complexity of non-DSB clustered lesions, suggesting that the complexity of DSBs converted from non-DSB clustered lesions may also increase.

## MATERIALS AND METHODS

### Monte Carlo damage simulation

The Monte Carlo damage simulation (MCDS) method provided estimates of the yield of clustered damage in a cell irradiated with photons, monoenergetic electrons, protons and heavy ions up to ^56^Fe ions [10, 30, 31]. In a constant target (cell nucleus) that had absorbed a dose of 1 Gy, the MCDS algorithm simulated the yields for different types of DNA damage. This algorithm employed reported DNA damage data and captured the major trends of DNA damage spectra from detailed track structure simulations. Because the damage yields simulated by the MCDS code implicitly accounted for DNA damage clusters caused by primary charged particles and secondary electrons in a typical mammalian cell, damage yields can be determined by weighting the yields by the fluence of primary charged particles. Types of DNA damage included BD, simple SSBs, simple DSBs, two or more strand breaks on the same strand (SSB^+^s), two or more strand breaks on the opposite strands but not constituting DSBs (2SSBs), DSBs with additional break(s) on a strand within 10 base pairs (DSB^+^s) and more than one DSB within 10 base pairs (DSB^++^s). The total SSBs referred to the combination of SSBs, SSB^+^s and 2SSBs. The total DSBs referred to the combination of DSBs, DSB^+^s and DSB^++^s. MCDS also provided estimates of DSBs in the presence of DMSO and adjusted them according to the fraction of non-scavengeable DNA damage (*FNSD*) and concentration at half-level (*CHMX*). *FNSD* represented the fraction of strand breaks and BD that were not scavengeable, and *CHMX* can be interpreted as the concentration of DMSO that reduced the amount of BD within the DNA segments by 50% [31]. MCDS can reasonably approximate experimental data for DSB yields by choosing the values 0.52 and 0.21 M for *FNSD* and *CHMX*, respectively, for ^60^Co gamma rays; and 0.75 and 0.14 M, respectively, for helium ions (3.31 MeV) [31].

### Monte Carlo excision repair simulation

The Monte Carlo excision repair (MCER) code was used to provide the probability of the repair outcomes in the BER and NER pathways for DNA damage that formed in the cells irradiated with electrons, protons and helium ions [22]. Prompt DSBs (formed directly by radiation) would be recorded only, and the non-DSB clusters that could be repaired through the BER and NER pathways were processed with MCER. The possible repair outcomes were correct repair, repair with a mutation, and conversion into a DSB. The third outcome arose from the misrepair of some sugars or BD, which converted these non-DSB clusters into DSBs. For example, an unrepaired strand break might be located on a site opposite a damaged base or an apurinic/apyrimidinic (AP) site where the base or AP site was a target for removal. During the removal, the DNA backbone is incised to form a break near an existing SSB, and an enzymatic DSB will be formed. MCER provided results specific to both SP BER and LP BER, as well as to NER pathways with a given set of parameters (see below). This model allowed for interactions between pathways and specified the relative contribution of each pathway to the overall repair of DNA damage. The definitions and roles of these input parameters are explained elsewhere [22]. To generate the MCER results, we used the following parameters for the input condition: inhibition distance = 8 base pairs; probability of choosing a lesion from the first strand break = 0.5; polymerase error rate for SP BER = 1.0^−4^; polymerase error rate for LP BER and NER = 1.0^−6^; probability of incorrect insertion opposite a damaged base = 0.75; probability of incorrect insertion opposite a lost base = 0.75. As suggested, repair outcomes when DMSO was present in the cell medium were calculated with the values 0.52 and 0.21 M for *FNSD* and *CHMX*, respectively, for ^60^Co gamma rays; and 0.75 and 0.14 M, respectively, for helium ions (3.31 MeV) [31].

### Calculation of DSB conversion from DNA damage

The DSB conversion for helium ions was calculated by using the formula that enzymatic DSB = $\sum_{i}\;p_{i}(E)Y_{i}(E)$ . *p_i_* (*E*) was defined as the conversion probability of repair pathways for DNA damage clusters composed of *i* lesions, and *Y_i_* was the yield of total non-DSB clusters per Gy per gigabase pairs (per Gy per Gbp) composed of *i* lesions with helium ions of energy *E*. The enzymatic DSBs for ^60^Co gamma rays was calculated by using the formula for dose-weighted DSBs [32]:

(1) $$Y_{i}=\frac{\int_{0}^{\infty }dEY_{i}(E)\;p_{i}(E)\Phi (E)\text{LET}{\;}_{\infty }(E)}{\int_{0}^{\infty }dE\Phi (E)\text{LET}{\;}_{\infty }(E)},$$

where Φ was the total fluence of secondary electrons produced in the cell medium through interactions of ^60^Co photons, and Φ(*E*) was the energy fluence of secondary electrons. For electrons with energies higher than 1 keV, the unrestricted LET (stopping powers) from the National Institute of Standards and Technology was used [33].

### Oxygen enhancement ratio

The biological effects of oxygen concentrations on cells can be quantified in terms of the OER, the ratio of hypoxic dose to aerated dose needed to achieve the same biological effects [9]. OER may also be defined as the ratio of biological effects such as DSB yields or cell killing at the same dose [32]. Here, OER was defined as the ratio of the yield of non-DSB clusters under aerobic conditions (21% O_2_) to that under anoxic conditions (2% O_2_).

## RESULTS

To gain insight into the effects of indirect action on DNA damage, the results for various types of DNA damage in the presence and in the absence of DMSO are summarized in Table 1. Table 1 shows that indirect action has a significant impact on DNA damage yields of cells irradiated with ^60^Co gamma rays and helium ions. Total damage due to ^60^Co gamma rays in the presence of DMSO decreased by 35%, whereas 2SSBs and DSB^+^s decreased by ∼80%. Total damage due to helium ions did not decrease, but the constituents of DNA damage changed. In the presence of DMSO, BD and SSBs increased by 20% and 3%, respectively. However, the yields of other types of complex damage, namely, 2SSBs, DSB^+^s and DSB^++^s decreased by 44%, 40% and 63%, respectively. These data suggest that indirect action affected the constituents of the complexity of DNA lesions induced by low- or high-LET radiation, contributing significantly only to the DSB yields for low-LET radiation.

**Table 1.** Absolute yields of DNA damage induced by ^60^Co gamma rays and helium ions (LET = 120 keV/μm) in the absence or presence of 2 M DMSO

Absolute yields (per Gy per Gbp)	BD	SSB	SSB^+^	2SSB	DSB	DSB^+^	DSB^++^	Total SSB	Total DSB	Total damage
^60^Co	422.48	177.83	7.93	0.98	7.07	0.95	0.11	187.37	8.14	617.36
^60^Co + DMSO	287.06 (32%↓)	109.44 (38%↓)	2.61 (67%↓)	0.18 (82%↓)	2.50 (65%↓)	0.18 (81%↓)	0.01 (91%↓)	112.73 (40%↓)	2.69 (67%↓)	401.99 (35%↓)
Helium ions	113.88	81.88	19.02	8.17	11.14	7.07	5.91	109.01	24.12	247.06
Helium ions +DMSO	137.13 (20%↑)	84.74 (3%↑)	13.72 (28%↓)	4.57 (44%↓)	9.12 (18%↓)	4.21 (40%↓)	2.16 (63%↓)	102.97 (6%↓)	15.49 (36%↓)	255.65 (3%↑)

As described above, it showed that DNA damage included base damage (BD), simple single-strand break (SSB), simple double-strand break (DSB), two or more strand breaks on the same strand (SSB^+^), two or more strand breaks on the opposite strands but not constituting DSB (2SSB), DSBs with additional break(s) on a strand within 10 base pairs (DSB^+^) and more than one DSB within 10 base pairs (DSB^++^).

**Fig. 1.**
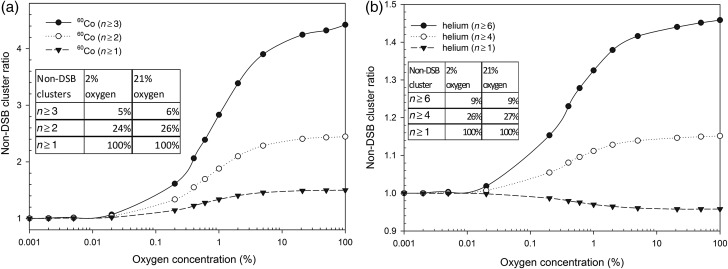
The non-DSB cluster ratio for ^60^Co gamma rays and helium ions versus oxygen concentration by MCDS. The ratio of non-DSB clusters was the ratio of the total yield of clustered DNA damage under the chosen aerobic condition to that under anoxic condition (0% O_2_). The table (inset) summarizes the percentages of various numbers of lesions per cluster in all non-DSB clusters. (**a**) Ratio of number of lesions per cluster composed of three or more (*n* ≥ 3, solid line), two or more (*n* ≥ 2, dotted line), and one or more lesions (*n* ≥ 1, dashed line) in cells exposed to ^60^Co gamma rays versus oxygen concentration. (**b**) Ratio of number of lesions per cluster composed of six or more (*n* ≥ 6, solid line), four or more (*n* ≥ 4, dotted line), and one or more lesions (*n* ≥ 1, dashed line) in cells exposed to helium ions versus oxygen concentration.

Oxygen also played an important role in DNA damage yields. MCDS results showed that DSB induction by ^60^Co gamma rays was 6.84 per Gy per Gbp under anoxic conditions (2% O_2_, cellular conditions [1]) and 8.13 per Gy per Gbp (1.2-fold) under aerobic conditions (21% O_2_, atmospheric conditions). The amount of total DNA damage induced by ^60^Co gamma rays increased by up to 6% going from 2% O_2_ to 21% O_2_; unsurprisingly, the yields of non-DSB clusters also increased. However, the ratio of non-DSB clusters varied with the number of lesions within a cluster (Fig. 1a). The ratio of non-DSB clusters was the ratio of the total yield of clustered DNA damage under the chosen aerobic conditions to that under anoxic conditions (0% O_2_). The ratio of non-DSB clusters due to ^60^Co gamma rays composed of three or more lesions (*n* ≥ 3) was more sensitive to the oxygen concentration than the ratio of clusters composed of two or more lesions (*n* ≥ 2) and the ratio of the total non-DSB clustered damage (*n* ≥ 1; one or more lesions in a cluster). The OER for non-DSB clusters composed of three or more lesions (*n* ≥ 3) was ∼1.3, whereas that for simpler clustered damage (*n* ≥ 2) was ∼1.15, highlighting the importance of oxygen in cluster complexity. MCDS predicted that clusters composed of two or more lesions (*n* ≥ 2) comprised 26% of the total non-DSB clusters and that clusters composed of three or more lesions (*n* ≥ 3) comprised 6% at 21% O_2_, indicating that most clusters were composed of one or two lesions. In contrast to those due to ^60^Co gamma rays, the total DNA damage induced by helium ions remained constant (∼250 per Gy per Gbp), and the yields of DSB induction were also approximately invariable (from 23.5 to 24.1 per Gy per Gbp) when the O_2_ concentration increased from 2% to 21%. Moreover, the OER of non-DSB clusters composed of six or more lesions (*n* ≥ 6) or less-complex damage (*n* ≥ 4) induced by helium ions (in Fig. 1b) was generally ∼1. Clusters composed of four or more (*n* ≥ 4) and six or more lesions (*n* ≥ 6) comprised 27% and 9% of total non-DSB clusters at 21% O_2_, and non-DSB cluster ratios increased as the oxygen concentration increased. However, the complexity of non-DSB clusters induced by high-LET radiation was less sensitive to the change in oxygen concentration.

Table 2 shows the probability of correct repair, mutation and enzymatic DSBs in the LP BER pathway in cells exposed to ^60^Co gamma rays and helium ions (LET = 120 keV/μm). As expected, the repair outcomes for cells irradiated with ^60^Co gamma rays were more favorable than those for cells irradiated with helium ions, as most of the damage induced by ^60^Co gamma rays was BD (see Fig. 1a and Table 1). The repair outcome for SSBs arising from ^60^Co gamma rays and helium ions was always poorer than that for BD and total damage. Misrepair of BD did not result in production of enzymatic DSBs. We obtained similar results for the other three pathways (SP BER, NER/SP BER and NER/LP BER); the probabilities of total damage only are shown in Tables 3–5.

**Table 2.** Repair outcome probabilities for SSB, BD, and total damage due to LP BER of cells irradiated with ^60^Co gamma rays and helium ions (LET = 120 keV/μm)

	Probability of correct repair		Probability of mutation		Probability of DSB conversion	
Damage type	^60^Co	Helium ions	^60^Co	Helium ions	^60^Co	Helium ions
SSB	0.93	0.66	0.04	0.21	0.03	0.13
BD	0.98	0.89	0.02	0.12	0.00	0.00
Total damage (BD + SSB)	0.96	0.78	0.03	0.16	0.01	0.06

Effects of the radical scavenger DMSO on repair outcomes for DNA damage due to ^60^Co gamma rays and helium ions are shown in Tables 3–4. Probabilities of correct repair of damage due to ^60^Co gamma rays were ∼90% or above and those of mutation and DSB conversion were in the ranges of 1–6% and 1–4%, respectively (Table 3). Probabilities of correct repair of damage due to helium ions were ∼58–89% and those of mutation were ∼6–23% (Table 4). Probabilities of DSB conversion were 5–20%, suggesting that the DSB conversion from non-DSB clusters might be an important source of DSBs. Depending on repair pathways, DMSO improved the probability of correct repair of DNA damage induced by ^60^Co gamma rays by 1–5% and reduced the probability of mutation and DSB conversion by 50–60%. These results indicated that indirect action had a strong effect on repair of DNA damage due to gamma rays. Similarly, DMSO improved the probability of correct repair of DNA damage induced by helium ions by 5–19% and reduced the probability of mutation and DSB conversion by 60–80% (Table 4). Repair outcomes via the SP BER pathway were always better than those via the LP BER pathway or the NER/BER pathways. For the NER/BER pathways, the length of patch had little impact on the repair outcomes. Repair probabilities for the LP BER pathway were likely to be between those of the SP BER and the NER/BER pathways.

**Table 3.** Repair outcome probabilities averaged over all types of DNA damage of cells irradiated with ^60^Co gamma rays in the absence or presence of 2 M DMSO

	Probability of correct repair		Probability of mutation		Probability of DSB formation	
Repair scenario	^60^Co	^60^Co + DMSO	^60^Co	^60^Co + DMSO	^60^Co	^60^Co + DMSO
SP/BER	0.98	0.99	0.01	0.01	0.01	0.00
LP/BER	0.96	0.98	0.03	0.02	0.01	0.01
NER/SP BER	0.90	0.94	0.06	0.04	0.04	0.02
NER/LP BER	0.90	0.94	0.06	0.04	0.04	0.02
Range	0.90–0.98	0.94–0.99	0.01–0.06	0.005–0.04	0.01–0.04	0.00–0.02

**Table 4.** Repair outcome probabilities averaged over all types of DNA damage of cells irradiated with helium ions (LET = 120 keV/µm) in the absence or presence of 2 M DMSO

	Probability of correct repair		Probability of mutation		Probability of DSB formation	
Repair scenario	Helium ions	Helium ions +DMSO	Helium ions	Helium ions +DMSO	Helium ions	Helium ions +DMSO
SP/BER	0.89	0.93	0.06	0.04	0.05	0.03
LP/BER	0.78	0.85	0.16	0.11	0.06	0.04
NER/SP BER	0.59	0.69	0.22	0.17	0.20	0.13
NER/LP BER	0.58	0.69	0.23	0.18	0.20	0.13
Range	0.58–0.89	0.69–0.93	0.06–0.23	0.04–0.18	0.05–0.20	0.03–0.13

**Fig. 2.**
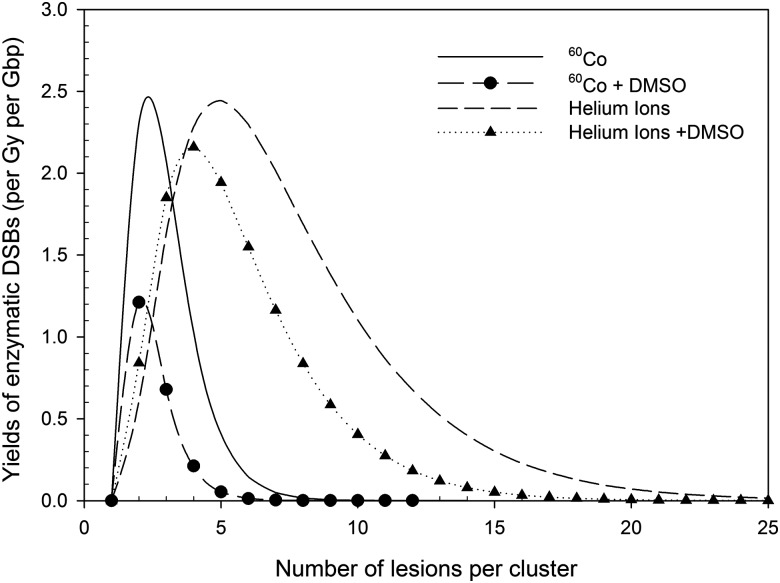
Yields of enzymatic DSB (per Gy per Gbp) in LP BER versus number of lesions per cluster. Yields induced by ^60^Co gamma rays were apparently lower than those induced by helium ions. Addition of DMSO reduced the yields induced by both ^60^Co gamma rays and helium ions.

To estimate the RBE for DSB conversion, we calculated the yields of enzymatic DSBs converted from the LP/BER pathway when cells were irradiated with ^60^Co gamma rays and helium ions (Table 5). In the presence of DMSO, yields of DSB induction for low- and high-LET radiation decreased by 66% and 36%, respectively. RBE values for DSB induction and maximum DSB conversion of helium ions (LET = 120 keV/μm) to ^60^Co gamma rays were 3.0 and 3.2, respectively. The respective increase in these values to 5.8 and 5.6 in the presence of DMSO indicated that indirect action contributed significantly to the RBE results. The yields of DSB conversion for both ^60^Co gamma rays and helium ions were potentially comparable with those of DSB induction. In addition, constituents of enzymatic DSBs (Fig. 2) due to low-LET radiation differed from those due to high-LET radiation. With low-LET radiation, the greatest number of lesions within an enzymatic DSB was less than five (peak was approximately two to three). Addition of DMSO reduced the DSB yields, without altering the number of lesions within a cluster (peak at two). DMSO scavenged some of the lesions within a cluster due to high-LET radiation, thereby shifting the peak from seven to five. DMSO reduced not only the DSB yields but also the number of lesions within a DSB, suggesting that indirect action contributed to the complexity of converted DSBs due to high-LET radiation.

**Table 5.** RBE of DSB induction and enzymatic DSB in LP BER of cells irradiated with ^60^Co gamma rays and helium ions (LET = 120 keV/µm) in the absence or presence of 2-M DMSO

Yields for DSB induction and enzymatic DSB in LP BER (per Gy per Gbp)					RBE for DSB induction and enzymatic DSB in LP BER			
	^60^Co	^60^Co +DMSO	Helium ions	Helium ions +DMSO	^60^Co	^60^Co +DMSO	Helium ions	Helium ions +DMSO
DSB induction	8.1	2.7 (67%↓)	24.1	15.5 (36%↓)	1.0	0.3 (67%↓)	3.0	1.9 (37%↓)
Maximum of DSB conversion	6.0	2.2 (64%↓)	19.1	12.1 (37%↓)	1.0	0.4 (64%↓)	3.2	2.0 (37%↓)

## DISCUSSION

In this study, we focused on the effects of indirect action and oxygen on DNA damage induction and on repair of damage due to low- and high-LET radiation. Indirect action of low-LET radiation contributed to the damage yields and the complexity of DNA damage, whereas that of high-LET radiation mainly correlated with the complexity of DNA damage, not the yields. Through the BER and NER pathways resulting from low- and high-LET radiation, DMSO reduced the contribution of indirect action. This reduction lowered the probability of misrepair and the number of the enzymatic DSBs, and increased the probability of correct repair. Moreover, the yields of DSB conversion were potentially comparable with those of DSB induction, indicating that enzymatic DSBs can have significant detrimental biological consequences.

Results of MCDSs of DSB induction by low- and high-LET radiation have been compared elsewhere [10, 30, 31]. MCDS results revealed that the DSB yields for cells irradiated with ^60^Co gamma rays in the presence of 2-M DMSO decreased to 60% (see Table 1). Similarly, an experimental study has observed that the DSB yields (induced by X-rays) decrease to 56% [34]. The MCDS has been shown to reproduce a spectrum for DNA damage similar to spectra obtained by detailed track structure simulations or experimental data for low- and high-LET radiation or for various oxygen concentrations. It can also reasonably approximate the DSB yields experimentally obtained at various concentrations of DMSO [10, 30, 31]. Interestingly, the MCDS predicted that the total damage due to helium ions (LET = 120 keV/μm) in the presence of DMSO did not decrease, and that BD and SSBs increased by 20% and 3%, respectively. These results suggest that DMSO can only reduce the complex damage to simpler damage but not the total yields resulting from high-LET radiation. This limitation could be attributed to the structure of high-LET radiation tracks. The track contains a core region and a penumbra. The core region of the track cannot be protected by DMSO because the core is mainly generated by direct action, while the penumbra is generated by very-low-energy electrons (delta rays) stopping close to the primary trajectory [35, 36]. OH radicals generated by low-energy electrons in high-LET radiation have ∼12% or higher probability of producing strand breaks [37]. Therefore, the OH scavenger DMSO can efficiently protect against the effects of delta rays and reduce complex lesions such as 2SSBs or DSBs to simpler damage (BD and SSBs).

In fact, oxic conditions are tightly correlated with indirect effects. DNA damage caused by indirect actions is reparable, but is permanent and irreparable if oxygen is present [1]. Hence, the damage is mainly caused by direct action of high-LET radiation, and is less affected by the effects of oxygen. Both direct and indirect effects of low-LET radiation contribute to DNA damage yields. Oxygen is required to convert indirect damage to lethal lesions [38, 39]. Studies indicate that damage yields, cluster complexity and cell death tend to decrease as oxygen concentration decreases [34, 40–42]. Moreover, the DSB conversion has been shown to be a function of oxygen concentration [10]. In our study, MCDS-derived results (Fig. 1) showed that the OER for gamma rays was 1.15–1.3, whereas that for helium ions was ∼1, depending on the cluster complexity. The OER was ∼1.1 for the induction of non-DSB (Fpg and Endo III) clusters in Hela cells exposed to 5 Gy of ^137^Cs gamma rays, which was in good agreement with the MCDSs [10]. As the LET increased, the OER decreased. This is probably due to the high spatial density of radicals and the apparent irrelevance of oxygen fixation or chemical repair to the damaged sites [43]. Nevertheless, clusters composed of higher lesion numbers are more sensitive to changes in oxygen concentration, suggesting a possible application in ion therapy. That is, if we seek to increase the dose to hypoxic tumor regions by a factor of OER to reach the same level of tumor control as in aerobic regions [9], we may need to consider the OER for higher cluster complexity, as complex damage may have more detrimental effects or ability to lead to cell killing [44].

MCER predicted that DMSO reduced the mutation frequency for cells irradiated with ^60^Co gamma rays by ∼50–60% (Table 3). This range agreed well with measurements of 70% for cells that received X-irradiation [45]. However, about 1–4% of total damage converted into enzymatic DSBs due to gamma rays, which was slightly lower than the experimental estimate of 10% [46]. The discrepancy between the MCER and experimental data was probably due to conversion of damaged sites containing heat-labile sites into DSBs; hence, the 10% estimate accounts for an artifact from the preparation of genomic DNA for pulse field gel electrophoresis (PFGE) [46–48]. If the artifact is excluded, the enzymatic DSB yields would decrease by a factor of 24%, giving a final value of 7% [49]. The difference between the MCER and the experimental estimate was small. Conversely, in the same study by Gulston *et al.* (2004), cells irradiated with helium ions did not have enzymatic DSBs, whereas MCER predicted that up to 5–20% of total damage converted into enzymatic DSBs. The difference in measured and predicted DSB yields may be ascribed to the loss of small fragments in the PFGE assay for high-LET radiation, and hence the yields of DSBs were not accounted for in PFGE assays [50, 51].

Table 5 indicates RBE values of 3.0 for DSB induction of helium ions to ^60^Co gamma rays and RBE values of 4.0–5.3 for cell survival [5]. The difference in RBE values between DSB induction and cell survival suggests that other factors were involved in addition to DSB induction, such as enzymatic DSBs converted from misrepair of DNA clustered damage [12]. Experimental studies have shown that the amount of clustered damage due to the treatment of different enzymes is comparable with prompt DSB induction [52]. Furthermore, the complexity of DSBs has been demonstrated as highly correlated with cell lethality [53]. Figure 2 shows that enzymatic DSBs caused by high-LET radiation are more complex than those caused by low-LET radiation, indicating that the lethality of high-LET radiation could be partially ascribed to the misrepair of non-DSB clusters.

Our results showed that the complexity of non-DSB damage had profound effects on DNA repair. Table 2 indicates that BD misrepair does not result in enzymatic DSBs, whereas the processing of complex SSB damage causes DSB conversion and elevates the incidence of mutation. However, a hierarchy of repairs minimizes DSB formation. SSBs significantly lower the rate of excision of some oxidative BD, such as 8-oxo-7,8-dihydroguanine (8-oxoG), until the SSBs are repaired, thus limiting the formation of DSBs [54–58]. This hierarchy extends the lifetime of non-DSB clustered damage induced in mammalian cells [59, 60], increasing the chance of clustered damage meeting a replication fork and thus producing a replication-induced DSB [61, 62]. DSBs produced through delayed repair of non-DSB clusters can further impair the processing of non-homologous end joining of the DSB induced directly by ionizing radiation [63]. The ultimate biological consequence of enzymatic DSBs could be catastrophic, as they are highly mutagenic or cytotoxic [24]. If this consequence is detrimental to normal cells, then this can be used as a tool for killing tumor cells [24, 64].

In summary, this study shows that the degree of complexity of DNA damage induced by high-LET radiation is significantly higher than that induced by low-LET radiation. Rates of mutation and DSB conversion from misrepaired non-DSB clusters induced by high-LET radiation are also higher than those arising from low-LET radiation. These rates can be greatly reduced with radical scavengers such as DMSO. Additionally, oxygen increases the complexity of non-DSB clustered lesions arising from both low- and high-LET radiation. Taken together, these results suggest that indirect action contributes significantly to the yields and complexity of DSBs converted during repair of non-DSB clusters due to high-LET radiation and can be used as tools in further radiotherapy.

## FUNDING

This research was supported by grants CMRPD1C0642&0662, CIRPD0061 and MOST103-2314-B-182-062 to F.-H.C. Funding to pay the Open Access publication charges for this article was provided by MOST103-2314-B-182-062.
